# PARP inhibition sensitizes childhood high grade glioma, medulloblastoma and ependymoma to radiation

**DOI:** 10.18632/oncotarget.362

**Published:** 2011-12-15

**Authors:** Dannis G. van Vuurden, Esther Hulleman, Olga L.M. Meijer, Laurine E. Wedekind, Marcel Kool, Hendrik Witt, Peter W. Peter Vandertop, Thomas Würdinger, David P. Noske, Gertjan J.L. Kaspers, Jacqueline Cloos

**Affiliations:** ^1^ Department of Pediatric Oncology / Hematology, Neuro-oncology Research Group, Cancer Center Amsterdam, VU University Medical Center, Amsterdam, the Netherlands; ^2^ Department of Molecular Genetics of Childhood Brain Tumors, German Cancer Research Center (DKFZ), Heidelberg, Germany; ^3^ Department of Pediatric Hematology and Oncology, Heidelberg University Hospital, Heidelberg, Germany; ^4^ Departments of Neurosurgery, Neuro-oncology Research Group, Cancer Center Amsterdam, VU University Medical Center, Amsterdam, the Netherlands; ^5^ Molecular Neurogenetics Unit, Department of Neurology, Massachusetts General Hospital and Harvard Medical School, Boston, MA, USA; ^6^ Department of Pediatric Oncology / Hematology, VU University Medical Center, Amsterdam, the Netherlands; ^7^ Department of Hematology, VU University Medical Center, Amsterdam, the Netherlands

**Keywords:** PARP, radiation, medulloblastoma, ependymoma, glioma, pediatric

## Abstract

Poly ADP-ribose polymerase (PARP) is a protein involved in single strand break repair. Recently, PARP inhibitors have shown considerable promise in the treatment of several cancers, both in monotherapy and in combination with cytotoxic agents. Synthetic lethal action of PARP inhibitors has been observed in tumors with mutations in double strand break repair pathways. In addition, PARP inhibition potentially enhances sensitivity of tumor cells to DNA damaging agents, including radiotherapy. Aim of this study is to determine the radiosensitizing properties of the PARP inhibitor Olaparib in childhood medulloblastoma, ependymoma and high grade glioma (HGG). Increased *PARP1* expression was observed in medulloblastoma, ependymoma and HGG, as compared to non-neoplastic brain tissue. Pediatric high grade glioma, medulloblastoma and ependymoma gene expression profiling revealed that high *PARP1* expression is associated with poor prognosis. Cell growth inhibition assays with Olaparib resulted in differential sensitivity, with IC_50_ values ranging from 1.4 to 8.4 μM, irrespective of tumor type and PARP1 protein expression. Sensitization to radiation was observed in medulloblastoma, ependymoma and HGG cell lines with subcytotoxic concentrations of Olaparib, which coincided with persistence of double strand breaks. Combining PARP inhibitors with radiotherapy in clinical studies in childhood high grade brain tumors may improve therapeutic outcome.

## INTRODUCTION

Tumors of the central nervous system (CNS) are the second most common type of pediatric cancer after hematologic malignancies. Malignant childhood brain tumors have an incidence of 3.2 per 100,000 persons, with a large heterogeneity of classifying diagnoses. Most commonly observed high grade childhood brain tumors are embryonal tumors (WHO grade IV medulloblastoma and supratentorial PNET), high grade astrocytic tumors (WHO grade III anaplastic astrocytoma and WHO grade IV glioblastoma multiforme (GBM)) and high grade ependymal tumors (WHO grade III anaplastic ependymoma) [1]. Treatment of children diagnosed with a high grade malignant CNS tumor consists of a multimodal approach based on surgery, radiotherapy and chemotherapy. Despite improved treatment strategies and a higher chance of survival, especially in standard risk medulloblastoma, mortality is still considerably high in high risk medulloblastoma, ependymoma and HGG [2-7].

Radiotherapy is an essential part of the treatment although notorious for induction of late effects, not only to the developing cortex and deep brain structures, but also to the posterior fossa, with higher risk when applied at younger age [8-13]. Radiosensitizers may offer the opportunity to ameliorate the therapeutic index by increasing the efficacy of radiotherapy, while reducing the toxicity and damage to the developing brain. The therapeutic index can for instance be improved by targeting specific characteristics of the tumors cells such as their replication dependency and DNA repair defects. In particular, inhibitors of DNA repair are potential treatment options in highly proliferative high grade childhood brain tumors, arising in largely non-replicative normal tissues with proficient DNA repair.

Poly ADP-ribose polymerase (PARP) enzymes have an essential role in single strand break (SSB) DNA repair and currently there are several clinically relevant PARP inhibitors available. Upon DNA damage, PARP1 is recruited to SSBs and the thereby induced PAR-chains, allow base-excision repair [14]. Moreover, PARP1 is involved in delaying the replication fork in homologous recombination (HR) proficient DNA damaged cells and in alternative pathways of non-homologous end joining (NHEJ) [15-17]. PARP inhibitors are in particular synthetically lethal in tumors with clear DNA repair deficiencies such as BRCA- tumors. However, both *in vitro* and mice studies indicate the rationale to combine PARP inhibitors with DNA damaging agents in many different tumor types. Recently, PARP inhibitor Olaparib was shown to increase radiosensitivity of non-small cell lung cancer *in vitro* and *in vivo*, by inhibition of DNA repair and reduction of hypoxia by increased tumor perfusion [18]. Moreover, PARP inhibitors are being investigated in phase I and II clinical trials for multiple cancers, as chemo- and radiosensitizers [19, 20]

A recent study indicated the high expression of *PARP1* in pediatric brain tumors, showing a significantly higher *PARP1* mRNA and protein expression in high-grade pediatric brain tumors, compared to their low-grade counterparts [21]. In addition, PARP1 protein expression was found in medulloblastoma patients including cell lines [22, 23]. These studies indicate that PARP1 may be a potential treatment target in these tumors. Therefore we investigated whether high *PARP1* expression is a biomarker of unfavorable prognosis in high grade pediatric CNS tumors. Moreover, we studied the rationale for combining PARP inhibitors with radiotherapy in patients with high PARP1 expression by determining the radiosensitizing properties of PARP1 inhibitors in these tumors using *in vitro* model systems.

## MATERIAL AND METHODS

### *In silico* analysis

R2, a microarray analysis and visualization platform, provided by the Department of Human Genetics of the Academic Medical Centre, Amsterdam, The Netherlands (http://r2.amc.nl), was used to obtain an overview of *PARP1* mRNA expression in high grade pediatric brain tumors. MAS5.0 normalized datasets of childhood HGG (n=53; GSE19578) [24], medulloblastoma (n=62; GSE10327) [25] and ependymoma (n=19; GSE13267) were compared to normal prefrontal cortex (n=44; GSE13564) [26] and normal cerebellum (n=9; GSE3526) [27]. Clinical relevance of PARP expression was evaluated in an independent ependymoma cohort (GSE27287)[28].

### Cell lines and cell culture

We used the pediatric brain tumor cell lines Res196 (fossa posterior ependymoma; Dr. Michael S. Bobola, Seattle Children's Hospital Research Institute)[29], SF188 and KNS42 (glioblastoma), UW479 (anaplastic astrocytoma), D283-med, D556-med (Dr. Darrell Bigner, Duke University) and UW228-2 (medulloblastoma). Cell lines were maintained in Dulbecco's Modified Eagle Medium (DMEM; PAA Laboratories GmbH, Pasching, Austria) containing stable glutamine and sodium pyruvate, supplemented with 1% penicillin/streptomycin (PAA Laboratories GmbH, Pasching, Austria) and 10% fetal bovine serum (FBS; PerBio Science Nederland B.V., Etten-Leur, The Netherlands). D283-med and D556-med cells were cultured in medium as above, without sodium pyruvate.

### Immunohistochemistry

Paraffin embedded 4 μm histological sections of 20 pediatric ependymomas (WHO grade II/III), 13 HGG (WHO grade III/IV) and a tissue microarray of 92 medulloblastomas were deparaffinized and rehydrated by washing the slides in a xylene and ethanol series. Sections were washed in water, endogenous peroxidase activity was quenched by a 30-minute incubation in 0.3% H^2^O^2^ in methanol, followed by washing in water. Antigen retrieval was performed with the sections in a 10 mM citrate buffer (pH 6.0) using a microwave oven. After washing in water and PBS, sections were incubated overnight at 4°C with previously validated mouse anti-PARP1 antibody (#556362; BD Pharmingen, San Diego, CA, USA), diluted 1:500 in normal antibody diluent (Immunologic, Duiven, The Netherlands). After thorough washing in PBS, sections were incubated for 15 minutes with post antibody blocking for PowerVision plus (Immunologic). Sections were subsequently rinsed in PBS and incubated with undiluted PowerVision plus Poly-HRP-anti Ms/Rb/Rt IgG (Immunologic) for 30 minutes and washed again in PBS. The antibody-peroxidase complex was detected by incubating the sections with 3,3'-diaminobenzidine solution (Envision-DAB, 1:50, DAKO, Glostrup, Denmark) for 10 minutes. The reaction was quenched in water. The slides were counterstained with hematoxylin, rinsed with ammonium water for color enhancement, dehydrated through a series of ethanol and xylene, and coverslipped.

### Western blotting

PARP1 protein expression was analyzed by Western blotting. Cells were lysed in homemade RIPA buffer (50mM Tris/HCL pH 7.5; 1% NP-40; 0.5% Na-deoxycholate; 150 mM NaCL; 0.05% SDS), supplemented with 1 mM Pefabloc SC (Roche Applied Science, Indianaoplis, IN, USA). Samples containing equal amounts of protein were separated on a 10% SDS–PAGE gel and blotted onto a PVDF membrane (Millipore, Amsterdam, The Netherlands). The membranes were blocked in TBS-Tween (20mM Tris; 137 mM NaCL pH 7.6; 0.1% Tween), containing 5% low fat dried milk and then incubated overnight at 4°C with an 1:3,000 diluted antibody directed against PARP1 (#556362; BD Pharmingen, San Diego, CA, USA) or with an 1:10,000 diluted antibody directed against β-actin (Santa Cruz Biotechnology, Santa Cruz, CA, USA). After several washes the membranes were incubated with 1:3,000 diluted HRP-conjugated rabbit-anti mouse IgG (DAKO, Glostrup, Denmark). Antibody binding was detected using Amersham ECL Plus Western Blotting Detection System (GE Healthcare, Buckinghamshire, UK).

### Cell proliferation assay

A cell proliferation assay was used to measure Olaparib (AZD2281 - Axon Medchem, Groningen, The Netherlands) drug sensitivity. Cells were plated at a concentration of 4,000 cells/ml for KNS42 and 2,000 cells/ml for SF188, UW479, Res196 and UW228-2 in 96-wells cell culture plates (Greiner Bio-One B.V., Alphen a/d Rijn, The Netherlands). After 24 hours, cells were treated with Olaparib (0 – 10 μM dissolved in DMSO). After 96 hours, plates were washed with PBS using a cell washer (AquaMax®4000, MDS Analytical Technologies Inc., Sunnyvale, CA, USA) and fixed with 4% formaldehyde (VWR International SAS, Fontenay-sous-Bois, France). After washing with PBS, cells were incubated for 30 minutes with DAPI (Sigma-Aldrich, Zwijndrecht, The Netherlands) diluted 1:3,000 in PBS using a reagent dispenser (Multidrop® Combi; Thermo Scientific, Waltham, MA, USA). Cells were counted using the Acumen eX3 laser scanning cytometer (TTP LabTech LTD, Melbourn, UK). Cell proliferation of in suspension growing medulloblastoma cell line D283-med was assessed using T25 culture flasks. Cells were seeded in 5 ml in T25 culture flasks, in a concentration of 50,000 cells/ml, in duplicate. After 24 hours, cells were treated with Olaparib (0 – 10 μM dissolved in DMSO). After 96 hours, cells were counted with a CASY® Cell Counter (Schärfe System, Germany).

To assess radiosensitizition with Olaparib, the cell proliferation assay using the Acumen eX3 laser scanning cytometer was used for KNS42 and Res196, and the CASY® Cell Counter for D283-med. At 24 hours after plating, Olaparib (0 – 8 μM) was added and cells were irradiated (0 – 6 Gy) 2 hours later, using a Gammacell 220 Excel (MDS Nordion, Ottowa, Canada). A 7-day growth curve was distilled from daily counts. Slope of the exponential growth was calculated per drug concentration, normalized to non-irradiated controls and plotted against radiation dose and analyzed using GraphPad Prism software (GraphPad Software Inc., San Diego, CA, USA). Analysis of inhibitory concentrations was performed using SigmaPlot 11.0 (Systat Software, Inc. San Jose, CA, USA). Triplicate experiments were performed for each cell line.

### Clonogenic assay

The radiosensitizing effect of PARP inhibition in Res196 cells was measured using a clonogenic assay. To assess clonogenic survival, exponentially growing Res196 cells were trypsinized, plated in triplicate in 6 wells plates at concentrations from 125/250 cells per well (0 Gy) to 2,000/4,000 cells (6 Gy) and allowed to attach for 4 hours. Olaparib (pre)treatment and radiation was applied as in the cell proliferation assay. Cells were grown for 10 days, fixed with 3.75% paraformaldehyde and stained with Giemsa stain modified solution (Fluka). Colonies of more than 50 cells were counted by an automated reader (Bioreader® 5000i; BIO-SYS, Karben Germany). This method was validated by a manual cell count giving comparable results. Plating efficiency (PE) was calculated by dividing the number of colonies counted by the number of cells plated. Surviving fractions (SF) were then calculated by dividing the PE by the PE of the non-irradiated control per drug concentration.

### Immunofluorescence for γH2AX foci detection

Cells were grown on coverslips and fixed in 4% methanol-free formaldehyde in PBS for 30 minutes, permeabilized with 0.1% Triton X-100 in PBS for 5 minutes, blocked with 5% FBS in PBS for 10 minutes and washed. Incubation with primary antibody anti-γH2AX (Millipore, Billerica, MA, USA) diluted 1:100 in PBS-gelatin was performed overnight at 4°C. Cells were washed four times and incubated with fluorescein-conjugated secondary antibody goat-anti-mouse antibody (1:200; DAKO, Glostrup, Denmark). Cells were rinsed thoroughly with PBS, treated for 5 minutes with DAPI diluted in PBS (1:10,000). Fluorescence was visualized with a Zeiss Axioskip microscope (HBO100W/Z), equipped with a Canon digital camera (Canon PowerShot A640, Canon Inc., Tokyo, Japan) as well as imaging software (Canon Utilities, ZoomBrowser Ex. 5.7, Canon Inc., Nort Ryde, Australia). The number of γH2AX foci was counted manually.

### Statistics

Survival data were available from pediatric medulloblastoma, ependymoma and HGG gene expression datasets[24, 25, 28], enabling the correlation between PARP1 expression and outcome. For HGG, cases that were a result of previous cranial radiation were excluded. The quantitative variable PARP1 expression was dichotomized according to the best cutoff value obtained by the receiver operating characteristics (ROC) analysis using the life status (dead vs alive) or the event of relapse (presence vs absence of relapse) as the outcome variable, respectively. Impact of high PARP1 expression on overall survival (OS) was determined using Kaplan Meier analysis and differences between groups were calculated using the log-rank test. Statistical tests were performed using SPSS software (version 15) and 2-sided calculated p values <0.05 were considered to be statistically significant.

## RESULTS

### *PARP1* mRNA expression is relatively high in pediatric brain tumors compared to normal brain tissue

**Figure 1:**
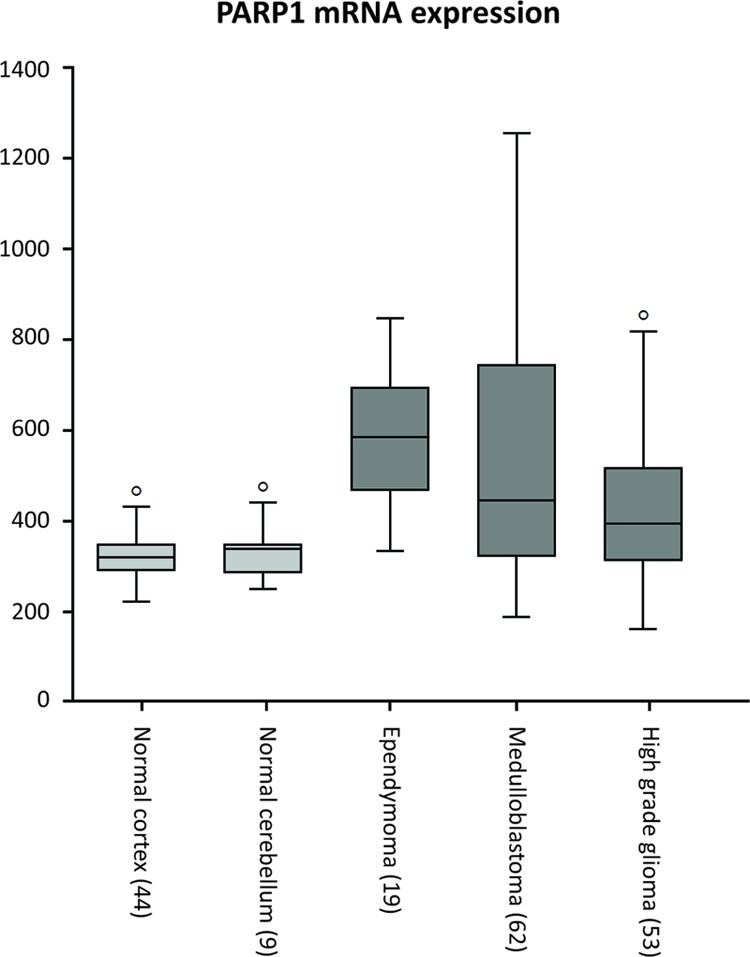
*In silico* analysis of *PARP1* mRNA expression using R2 analysis software on datasets of non-malignant brain tissues of cerebral cortex and cerebellum (grey), versus datasets of childhood ependymoma, medulloblastoma and high grade glioma (dark grey).

*PARP1* mRNA expression in pediatric brain tumors and non-malignant brain tissues was determined by *in silico* analysis of publicly available microarray data[24-27], using R2 analysis software. *PARP1* was found to be expressed in normal adult brain. Similar expressions were found for adolescent dorsolateral prefrontal cortex consisting of neuronal and glial cells and non malignant cerebellum. The *PARP1* expression was significantly higher in pediatric ependymoma and medulloblastoma, compared to cortex and cerebellum, while *PARP1* expression in HGG was significantly higher compared to normal cortex. (Figure 1).

### High *PARP1* mRNA expression is associated with poor outcome in pediatric HGG, medulloblastoma and ependymoma

For 55 medulloblastoma, 74 ependymoma and 38 pediatric HGG cases both microarray expression data and specific outcome data were available[24, 25, 28]. In order to determine the impact of PARP1 on survival, *PARP1* expression data were dichotomized on high versus low expression for each tumor type.

**Figure 2:**
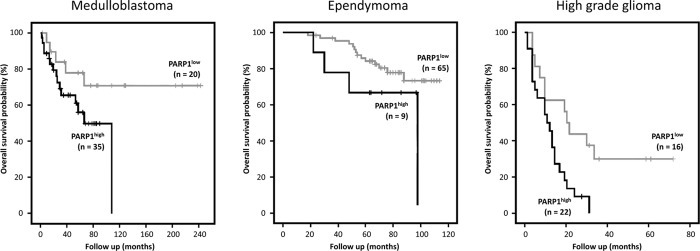
Kaplan Meier survival curves using R2 analysis software showing the overall survival probability for high (black) versus low (grey) *PARP1* mRNA expression, in patients with medulloblastoma, ependymoma and high grade glioma respectively High levels of *PARP1* showed a clear trend towards worse overall survival in medulloblastoma (*P*=0.073) and ependymoma (*P*=0.083) and were significantly correlated with a worse prognosis in pediatric HGG (*P*=0.009).

In the medulloblastoma dataset, in the PARP^high^ group (n = 35) 15 patients died (42.9%) while in the PARP1^low^ group (n = 20) 5 patients died (25%). Kaplan-Meier survival analysis showed a trend to worse overall survival in patients with high *PARP1* mRNA expression (n=28) (p=0.073). In the ependymoma dataset, in the PARP^high^ group (n = 9) 4 patients died (44.4%) while in the PARP1^low^ group (n = 65) 14 patients died (21.5%). Again a trend was observed in the Kaplan-Meier survival analysis to worse overall survival in patients with high *PARP1* mRNA expression (p=0.073). For pediatric HGG an even stonger association with PARP expression was observed. In the PARP^high^ group (n = 22) 21 patients died (95.9%) while in the PARP1^low^ group (n = 16) 11 patients died (68.7%). Kaplan-Meier survival analysis showed a significantly worse overall survival in pediatric HGG patients with high *PARP1* mRNA expression (p=0.009) (Figure 2).

### PARP1 protein expression in primary tumor samples

**Figure 3:**
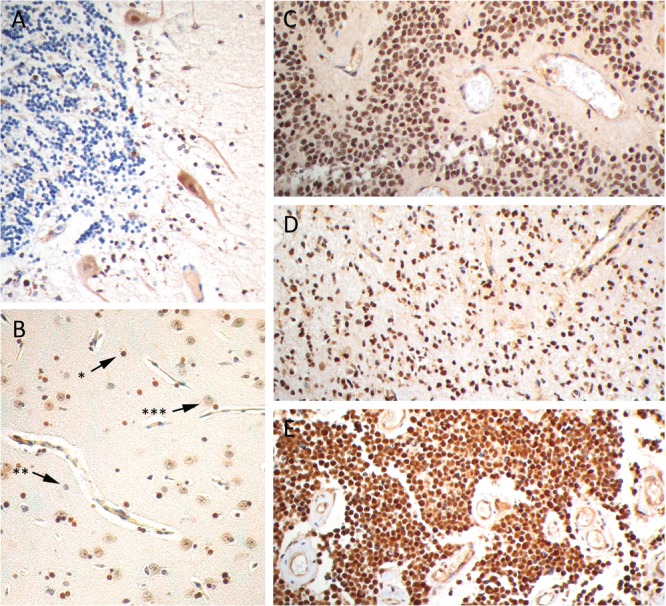
PARP1 immunohistochemistry on paraffin embedded sections Representative images of PARP1 staining in (A) cerebellum, (B) cerebral cortex, with strong nuclear staining of oligodendrocytes (*) and weak nuclear staining of astrocytes (**) and neurons (***), strong nuclear staining in (C) anaplastic ependymoma, (D) anaplastic astrocytoma and (E) medulloblastoma.

PARP1 protein expression was determined by immunohistochemistry in non-malignant brain tissues and pediatric CNS tumors (Figure 3). Non-malignant cerebellum showed cytoplasmic staining of Purkinje cells and weak staining of cerebellar neurons; granule cells were completely negative for PARP1. In normal neo-cortex only oligodendrocytes showed a strong positive nuclear staining for PARP1. Astrocytes and neurons had a weak PARP1 expression. The pediatric CNS tumors revealed a relatively strong PARP1 protein expression. Within tumor tissue sections from pediatric HGG, ependymoma and medulloblastoma patients, a comparable strong nuclear staining pattern was observed, which was scored semi-quantitatively based on intensity and percentage of positive cells. Results of this scoring system are summarized in table 1. In 7 out of 8 HGG tissue sections a strong to intensive nuclear staining was seen in at least 50% of cells. In tissue sections derived from ependymoma patients a similar tendency towards strong and abundant nuclear PARP1 staining was observed: in 16 out of 18 sections a convincing nuclear PARP1 staining was found in at least 30% of tumor cells. In medulloblastoma a more varied staining pattern was found, with a higher number of sections showing moderate nuclear PARP1 staining. Despite a larger variation in the percentage of positive cell nuclei, a majority of sections demonstrated positive nuclear PARP1 staining (61 out of 84 sections). Analogous to the mRNA expression, ependymoma samples show overall very high protein expression. Medulloblastoma and HGG are more variable but overall also highly express PARP1.

**Table 1:** Overview of semiquantitative scoring of immunohistochemical PARP1 staining in pediatric HGG, ependymoma, and medulloblastoma patient material The intensity of nuclear PARP1 staining (negative/moderate/strong/intensive) and the percentage of positive nuclei was determined by two independent reviewers (DVV/OM).

	Staining				
	% Positive cells	Negative	Moderate	Strong	Intensive
PEDIATRIC HGG (n=8)	< 10%	1	0	0	0
	10-50%		0	0	0
	50-90%		0	1	1
	> 90%		0	0	5
Ependymoma (n=18)	< 10%	3	0	0	0
	10-50%		0	0	1
	50-90%		0	1	1
	> 90%		0	1	11
Medulloblastoma (n=84)	< 10%	8	0	1	0
	10-50%		2	3	1
	50-90%		12	11	5
	> 90%		1	15	25

### Pediatric brain tumor cell lines are differentially sensitive to PARP inhibition with Olaparib

**Figure 4:**
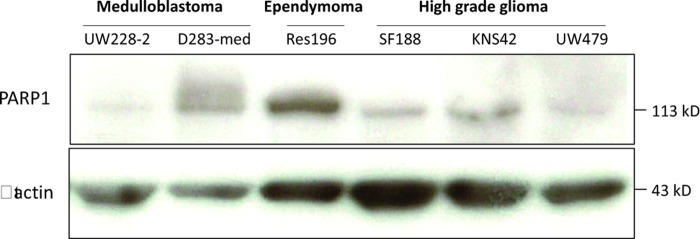
Immunoblot of baseline PARP1 (113 kD) and beta actin (42 kD) expression in pediatric brain tumor cell lines

We examined PARP1 protein expression in different pediatric brain tumor cell lines by Western Blot analysis (Figure 4). D283-med and UW228-2 medulloblastoma cells, Res196 ependymoma cells and SF188, KNS42 and UW479 HGG cells were used. In all cell lines PARP1 protein expression was detected, in particular in ependymoma cell line Res196 and medulloblastoma cell line D283-med (Figure 3).

**Figure 5:**
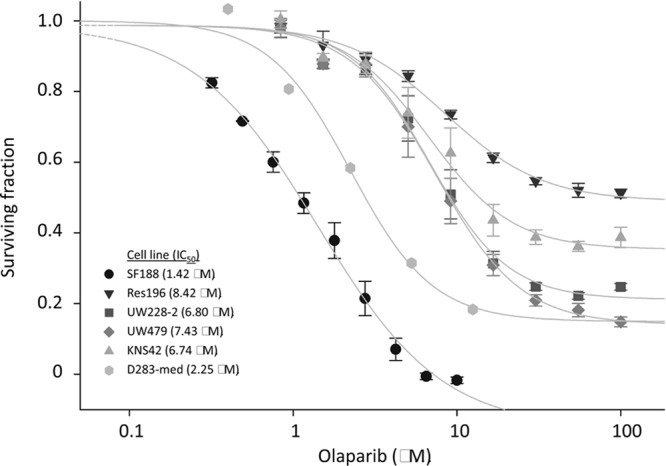
Cell viability assay for pediatric brain tumor cell lines, exposed to the indicated concentrations of Olaparib Cell viability was assayed after 96 h exposure. Dots indicate mean values of experiment performed in triplicate. Bars indicate standard error of the mean (SE). IC50 values are calculated using SigmaPlot and are indicated between brackets.

To determine the effect of PARP inhibition on cell proliferation we first exposed the cells to the PARP inhibitor Olaparib as a single agent. In figure 5 the effect of Olaparib treatment on six pediatric brain tumor cell lines is shown. The HGG cell line SF188 showed high sensitivity to Olaparib treatment as compared to the other cell lines, with a 50% inhibitory concentration (IC_50_) of 1.42 μM. The medulloblastoma cell line D283-med is also highly sensitive, with an IC_50_ value of 2.25 μM. HGG cell lines KNS42 and UW479 are less sensitive, with IC_50_ values of 6.74 μM and 7.43 μM respectively. A comparable sensitivity was observed in the medulloblastoma cell line UW228-2 for which an IC_50_ of 6.80 μM was found. Olaparib appeared to convey minimal toxicity in the ependymoma cell line Res196, with an IC_50_ value of 8.42 μM.

### PARP inhibition with olaparib sensitizes pediatric brain tumor cells to radiation

**Figure 6:**
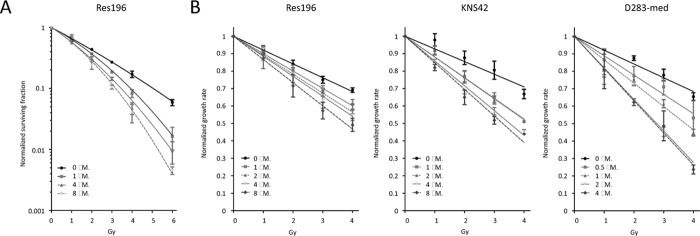
(A) Clonogenic survival of Res196 cells 10 days after irradiation (0 – 6 Gy). Cells were treated with different dose of Olaparib prior to irradiation. All treatment concentrations increased radiation sensitivity compared to the untreated control, (p < 0.001). A representative experiment is shown (n=3). (B) Growth rate of pediatric brain tumor cell lines after treatment with different doses of Olaparib and irradiation, normalized to non-irradiated control cells. A difference in slope of the exponential growth rate is a measure for radiosensitization.

To investigate whether a two hour pretreatment with Olaparib enhances radiosensitivity in ependymoma, HGG and medulloblastoma cell lines, clonogenic and cell proliferation assays were performed. Only ependymoma cell line Res196 was able to form clones in a clonogenic assay. Using this assay, Olaparib increased radiation sensitivity compared to the untreated control in a dose-dependent fashion (p < 0.001) (Figure 6A). In the absence of irradiation, clonogenic capacity was reduced with 12%, 18% and 40% in cells treated with 1, 4 and 8 μM Olaparib, respectively (data not shown). Radiosensitizing capacity of Olaparib was also investigated in Res196 cells by a previously described cell proliferation assay[30](Figure 6B). Like in the clonogenic assay, radiosensitization was observed in this assay: upon Olaparib treatment, a dose-dependent decrease of exponential growth after radiation was observed in Res196 cells (Olaparib IC50 8.42 μM, Figure 5), even in subcytoxic concentrations of 1, 2 and 4 μM.

Since the HGG cell line KNS42 and medulloblastoma cell line D283-med were not able to form clones, the cell proliferation assay was used in these cells. Results from the proliferation assay are shown in figure 6B. In HGG cell line KNS42 an even more profound effect was observed when cells were irradiated in the presence of Olaparib, indicating a stronger sensitizing effect towards radiation. Based on the toxicity data of Olaparib (Figure 5), showing an IC_50_ of 6.74 μM, treatment with 1 and 4 μM Olaparib showed the optimal radiation sensitizing effect in KNS42.

In medulloblastoma cell line D283-med, showing relatively strong sensitivity to Olaparib, with an IC_50_ of 2.25 μM doses of 0.5 μM to 4 μM were used in combination with radiation. Again radiosensitization was observed, even in subcytotoxic concentrations of 0.5 μM (Figure 5B).

### Pretreatment with Olaparib results in persistence of DNA damage after irradiation of pediatric brain tumor cells

**Figure 7:**
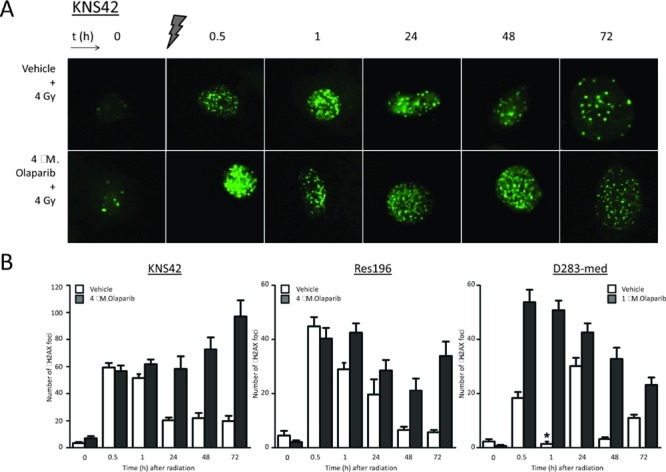
(A) Representative examples of immunofluorescent staining of γH2AX foci before, and (0.5 – 72 hours) after 4 Gy gamma radiation in KNS42 cells, pretreated with vehicle or 4 μM Olaparib. (B) Mean ± SE counts of γH2AX foci in KNS42, Res196 and D283-cells. D283-med cells were pretreated with 1 μM Olaparib. One hour after radiation nuclei stained diffusely positive for γH2AX in D283-med cells (*), though few foci could be detected.

In order to study the effect of PARP1 inhibition on the induction and persistence of DSBs in ependymoma, HGG and medulloblastoma cells, the presence of γH2AX foci was assessed by immunofluorescence. In HGG cell line KNS42, ependymoma cell line Res196 and medulloblastoma cell line D283-med, a rapid formation of γH2AX foci was observed minutes after ionizing radiation (Figure 7). A two hour pretreatment with 1 μM (D283-med), or 4 μM (KNS42, Res196) Olaparib did not result in a significant difference of baseline DSB levels compared to untreated cells before radiation. However, one hour after irradiation a significantly higher number of DSBs was observed in Olaparib pretreated cell line Res196 and D283-med, a difference that persisted even up to 72 hours after radiation (Figure 7B). Cell line KNS42 showed an increasing difference in DSB later in time, with γH2AX foci count still rising at 72 hours after irradiation in cells pretreated with Olaparib.

## DISCUSSION

Here we show that PARP1 is expressed in high grade pediatric brain tumors, thereby indicating the rationale of PARP1 as a potential therapeutic target in these cancers. *In silico* analysis of *PARP1* mRNA expression revealed higher *PARP1* expression in pediatric brain tumors compared to non-malignant brain. Interestingly, high *PARP1* mRNA expression was associated with poor disease outcome in a gene expression datasets of medulloblastoma, posterior fossa ependymoma and pediatric HGG, with high significance in the latter. As a validation of the gene expression data, we assessed PARP1 protein expression by immunohistochemistry in high grade pediatric brain tumors and tissues derived from non-malignant pediatric brain. Although subtypes of cells in non-neoplastic brain region showed some PARP1 positivity, an abundant and profusely intensive nuclear PARP1 staining was observed in tissues of pediatric brain tumors, especially HGG and ependymoma. Medulloblastoma tissue was more variable in PARP1 expression but compared to normal tissue it also generally showed strongly positive staining.

In all pediatric brain tumor cell lines used in this study, strong PARP1 protein expression was demonstrated by Western Blot.

The HGG cell line SF188, which showed a moderate PARP1 expression had a high sensitivity to Olaparib. Compared to the other, relatively resistant, HGG cell lines KNS42 and UW479 this intrinsic sensitivity to Olaparib was remarkable. It may be explained by the fact that the SF188 cell line has a focal deletion of the tumor suppressor gene NF1 (17q11.2) [31]. Inactivating NF1 deletions or mutations have recently been shown in 23% of adult glioblastoma patient samples and 6% of pediatric HGG [24, 32]. Impairment of NF1 tumor suppressor gene function leads to Ras/RAF/MEK/ERK and Ras/PI3K/AKT/PKB/mTOR pathway overexpression, thus affecting cell proliferation, growth and survival [33, 34]. Disturbances in these pathways indirectly impair DNA repair, which possibly explains the high sensitivity of SF188 to Olaparib [35]. Furthermore, in the study by Bax *et al.,* a point mutation in TP53 (17p13) was found in this cell line, impairing apoptosis and hampering G1 arrest [31]. Although Bax *et al* showed the HGG cell line KNS42 to harbor the same p53 mutation, KNS42 revealed a much lower sensitivity to Olaparib. The genetic profile of SF188 and its high sensitivity to Olaparib found in our study, suggests a cooperative effect of PARP inhibition on one side and loss of NF1 and TP53 on the other, leading to synthetic lethality. Xenograft studies have shown that NF1 inactivation in mutationally p53 inactivated mice induced malignant astrocytomas and secondary malignant tumors after radiation [36, 37]. Future research is warranted to determine if loss of NF1 indeed conveys specific sensitivity to PARP inhibition.

Defects in DSB repair in pediatric brain tumors, providing rationale of synthetic lethality with PARP1 inhibition are to be explored. Recently, low-level gains in PARP1 were identified in a subset of patients suffering from diffuse intrinsic pontine glioma (DIPG), using a whole-genome single nucleotide polymorphism (SNP) – based microarray. Multiple deletions or LOH in genes involved in HR, such as BRCA1/2, RAD50, RAD51L1, and in NHEJ, such as LIG4, XRCC4 and XRCC5, were observed in this SNP-array, making exploration of PARP inhibition in these tumors even more relevant. Furthermore, in DIPG, phosphatase and tensin homolog (PTEN) loss was observed in 8-57% in small case series [38-40]. Loss of PTEN through deletion or mutation in supratentorial pediatric HGG is more rare than in DIPG, but if present, associated with poor overall survival [41]. As a transcriptional regulator of RAD51, PTEN is involved in DSB repair and preservation of chromosomal integrity. Lack of PTEN function compromises homologous recombination through decreased expression of RAD51. In this respect, PTEN loss is described to be an indicator of PARP inhibitor sensitivity [42-45].

As in the HGG cell line SF188, an almost similar degree of sensitivity to treatment with Olaparib as a single agent was seen in the medulloblastoma cell line D283-med. Recently, a number of studies were conducted to gain more knowledge on distinct gene expression profiles of medulloblastoma, thereby improving the molecular classification of these tumors [25, 46-48]. Our data indicate that PARP1 inhibition is a potential treatment target for high-risk patients and this will be even more relevant in combination with radiation. In this study a significant radiation sensitizing effect of treatment with the PARP1 inhibitor Olaparib was demonstrated in medulloblastoma, HGG and ependymoma cell lines. For the evaluation of radiation sensitivity, two distinctive methods were used. The clonogenic assay is regarded as the gold standard for the determination of radiation sensitivity [49]. This method assesses the ability of neoplastic stem cells for self-renewal and regrowth after treatment, thought to be the major causes for treatment failure and tumor recurrence in patients. An alternative method to the clonogenic assay is the assessment of cell proliferation after irradiation, providing information on the proliferative capacities of surviving cells after radiation [30]. Here we show that Olaparib sensitizes ependymoma cell line Res196 to irradiation in both assays, supporting the use of the cell proliferation assay as a method to study radiation sensitization. This radiosensitizing effect was also observed in pediatric HGG cell line KNS42 and, to a larger extent, medulloblastoma cell line D283-med. Olaparib sensitized these cells in subcytotoxic concentrations, proving its virtue as a true radiosensitizing drug.

PARP1 is well known for its prominent role in the repair of SSBs. Meanwhile, its importance in DSB repair is gradually emerging [50, 51]. We assessed the effect of Olaparib on the preservation of DNA damage by visualizing γH2AX foci. All cells treated with Olaparib showed persistence of radiation induced DSBs, up to 72 hours after exposure to irradiation, whereas untreated cells were able to repair most of the damage. Persistence of DSBs in the Olaparib treated cells can be explained by two distinct mechanisms. First, impairment of SSB repair by PARP1 inhibition and consequent collapse of the replication fork leads to the formation of DSBs. The observed accumulation of yH2AX foci in KNS42 cells might be explained by this phenomenon. Alternatively, inhibition of PARP1 is suggested to lead to the binding of Ku to radiation induced DSBs, thereby preventing DSB repair by HR [52].

Tolerability and efficacy of PARP1 inhibitor Olaparib in cancer treatment has now been confirmed in adult phase I and II clinical trials [53, 54]. Since the use of PARP1 inhibitors in cancer treatment is still in an experimental phase, the long-term effects of PARP1 inhibition have not yet become apparent. Currently, a clinical trial in the USA is exploring efficacy of oral PARP inhibitor Veliparib (ABT-888) in combination with temozolomide in pediatric patients with recurrent or refractory CNS tumors [19].

Especially in the treatment of childhood cancer, long term-effects should be taken into serious consideration. From this perspective it is of importance to get more insight in PARP1 expression and function in developing, non-malignant brain tissues. This is necessary to determine the therapeutic index of PARP1 inhibitors, especially when used in combination with other DNA damaging agents. Recently, studies have shown that inhibition of PARP1 could convey a specific therapeutic benefit for the central nervous system. Following acute oxidative stress, such as radiation, neurons are described to have excessive synthesis of PAR by overactive PARP-1, leading to NAD^+^/ATP depletion and subsequent neuronal cell death. PARP inhibition has therefore been opted as a neuroprotective strategy for cerebral ischemia and several neurodegenerative diseases, such as Parkinson's disease and ALS [55, 56].

In conclusion, our study provides evidence that PARP1 inhibition with Olaparib enhances radiation sensitivity of pediatric ependymoma, medulloblastoma and pediatric HGG cells *in vitro*, thereby providing the basis for further *in vivo* studies, aiming at clinical trials combining PARP inhibitors upfront with (chemo)radiotherapy. This is a very important finding since some radiation resistant pediatric brain tumors could profit from a sensitization drug like Olaparib. This will potentially lead to more effective therapies with the possibility to reduce radiotherapy, conveying a better therapeutic index, ultimately leading to better survival with a reduction of long term side effects.
